# Astrocyte Heterogeneity and Metabolic Reprogramming: Mechanisms Governing Retinal Ganglion Cell Damage in Glaucoma

**DOI:** 10.3390/cells15060487

**Published:** 2026-03-10

**Authors:** Yufei Hao, Dongran Liang, Mengjie Ren, Fang Kuang, Mingmei Wu

**Affiliations:** 1 Department of Neurobiology, School of Basic Medicine, Fourth Military Medical University, Xi’an 710032, China; m19298217927@163.com (Y.H.); yuelaixiche@163.com (D.L.); r15639839293@163.com (M.R.); kuangf@fmmu.edu.cn (F.K.); 2 The Shaanxi Province Key Laboratory of Brain Function Analysis and Modulation, Xi’an 710032, China

**Keywords:** astrocyte heterogeneity, metabolic reprogramming, glaucoma, retinal ganglion cells, fatty acid metabolism, neuroinflammation, vision

## Abstract

Glaucoma, a leading cause of irreversible visual impairment, is driven by progressive retinal ganglion cell (RGC) degeneration. Emerging evidence highlights astrocytes as pivotal players in its pathogenesis, with their heterogeneity and pathological metabolic reprogramming profoundly impacting RGC survival. This review synthesizes current insights into astrocyte diversity and metabolic alterations during glaucoma-related RGC injury, emphasizing molecular mechanisms from proteomic studies. Key focuses include fatty acid metabolism, neuroinflammation, and signaling pathways that modulate astrocyte function and contribute to neurodegeneration. Despite advances, challenges remain—particularly in characterizing astrocyte subtypes and identifying actionable targets within astrocyte-mediated metabolic/inflammatory cascades. By unraveling the interplay between astrocyte heterogeneity, metabolic reprogramming, and RGC vulnerability, this review provides novel theoretical frameworks to inform targeted glaucoma therapies.

## 1. Introduction

Glaucoma is a leading cause of irreversible blindness worldwide, characterized by progressive degeneration of retinal ganglion cells (RGCs) and their optic nerve axons—the primary mediators of visual signal transmission. Affecting over 70 million people with rising prevalence linked to population aging [1], it features an insidious onset: substantial RGC loss typically precedes detectable visual impairment. Elevated intraocular pressure (IOP) is a major risk factor, yet a significant subset of patients develops normal-tension glaucoma, highlighting the condition’s multifactorial etiology. Current therapies, which focus solely on IOP reduction, fail to halt or reverse RGC degeneration, underscoring the urgent need to elucidate the molecular and cellular mechanisms of glaucomatous neurodegeneration for novel disease-modifying interventions [2].

Astrocytes, the most abundant glial cell population in the central nervous system (CNS)—including the retina—are indispensable for maintaining tissue homeostasis, supporting neuronal function, and mediating cellular responses to injury or pathological stress [3,4]. Mounting evidence now positions retinal astrocytes as key regulators of glaucoma pathogenesis: under physiological conditions, these cells collaborate with Müller glia to sustain neuronal viability, modulate oxidative stress, preserve blood-retinal barrier (BRB) integrity, and coordinate synaptic and metabolic crosstalk with RGCs [5]. In response to glaucomatous insults, astrocytes undergo a dynamic phenotypic shift termed reactive astrogliosis, marked by profound alterations in morphology, molecular expression, and functional properties. Notably, this reactivity is context-dependent, enabling astrocytes to exert either neuroprotective effects that mitigate RGC injury or detrimental actions that exacerbate neurodegeneration [6].

Recent breakthroughs in single-cell omics technologies have uncovered extensive heterogeneity in astrocyte morphology and function across CNS regions and disease states. Distinct subpopulations of retinal and optic nerve astrocytes exhibit divergent capacities to support axonal integrity and respond to injury [7,8]. Under pathological conditions, reactive astrocytes can be further subclassified into neuroprotective and neuroinflammatory subtypes, whose differential activation directly impacts RGC survival [9,10]. The development of novel tools for labeling and targeting specific astrocyte subtypes has provided unprecedented insights into their roles in neurodegenerative disorders such as glaucoma, opening new avenues for subtype-specific therapeutic exploration [11].

Metabolic reprogramming has emerged as another critical mechanism governing astrocyte responses to CNS injury, including glaucomatous damage [12]. As central orchestrators of retinal metabolic homeostasis, astrocytes regulate glucose and lipid metabolism, cholesterol turnover, and reactive oxygen species detoxification—processes that are profoundly dysregulated in neurodegenerative states [3,13]. In the early stages of injury, adaptive metabolic shifts in astrocytes may support RGC survival by supplying alternative energy substrates, restoring redox balance, and preserving synaptic function. Conversely, persistent or maladaptive metabolic reprogramming can drive chronic neuroinflammation and oxidative stress, ultimately accelerating RGC death [12].

High-throughput technologies including proteomics and single-cell RNA sequencing have revolutionized our understanding of the retinal microenvironment in health and disease, unraveling intricate molecular networks and intercellular crosstalk among astrocytes, neurons, and other glial cells in glaucoma [8,13,14]. These approaches have facilitated the systematic dissection of astrocyte activation cascades and regulatory mechanisms, laying the groundwork for the design of targeted therapies aimed at preserving RGCs and preventing vision loss.

**Figure 1:**
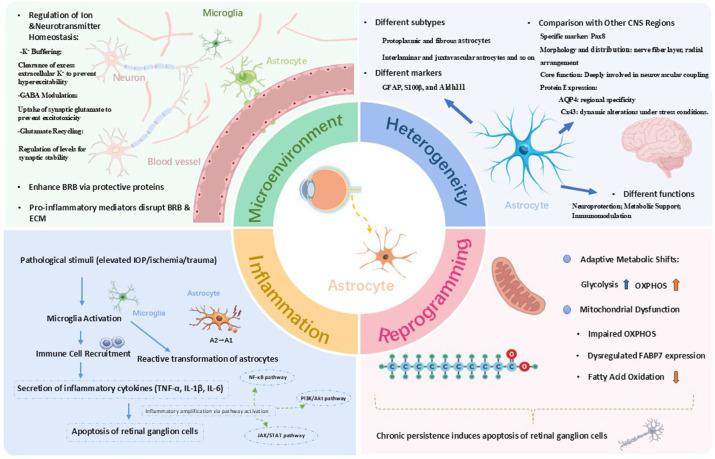
Astrocyte functions in glaucoma. This diagram depicts astrocyte functions in CNS homeostasis, inflammatory/metabolic reprogramming, and retinal ganglion cell degeneration. BRB: blood-retinal barrier; ECM: extracellular matrix; IOP: intraocular pressure; FAO: fatty acid oxidation.

In this review, we discuss the dual roles of astrocyte heterogeneity and metabolic reprogramming in modulating RGC survival, and explore the potential of targeting astrocyte subpopulations and metabolic pathways as a framework for next-generation glaucoma interventions to halt or reverse RGC degeneration (Figure 1). Finally, we summarized the effects of astrocyte responses on RGC in various glaucoma models in recent years (Table 1, Table 2 and Table 3).

## 2. Astrocyte Heterogeneity

### 2.1. Morphological and Molecular Marker Diversity

Astrocytes, the most abundant glial cells in the CNS, show significant diversity in morphology, distribution, and origin across species and brain regions. Traditionally classified into protoplasmic and fibrous types, recent studies reveal more specialized forms, including interlaminar and juxtavascular astrocytes, with distinct morphotypes in areas like the hippocampus [14,46,47,48]. Their heterogeneity is linked to developmental origins, as progenitor cells from various zones produce astrocytes with unique anatomical and molecular characteristics that persist into adulthood [49,50].

Astrocyte identification has relied on markers such as GFAP, S100β, and Aldh1l1. However, their variable expression limits specificity and captures only part of astrocyte diversity [51,52,53,54,55,56]. This has led to transcriptomic and proteomic methods revealing unique astrocyte subtypes with distinct gene profiles, influenced by region and disease, while astrocyte reactivity complicates their classification further [57,58].

### 2.2. Functional Heterogeneity

Astrocytes are increasingly recognized as fundamental to both physiological processes and the pathogenesis of neurodegenerative diseases such as glaucoma. This heterogeneity is evident not only in their morphological diversity but also in their distinct molecular signatures, electrophysiological properties, and region-specific functions [21]. In the context of the retina and optic nerve head—key sites for RGC vulnerability in glaucoma—astrocyte subtypes display divergent roles in neuroprotection, metabolic support, and inflammatory regulation [59]. Recent transcriptomic and proteomic studies have revealed that retinal and optic nerve astrocytes display distinct gene expression profiles and functional specializations. Pax8 serves as a specific marker for retinal astrocytes, while Cartpt remains specific for optic nerve head astrocytes following elevated IOP [60]. Astrocyte subpopulations dedicated to neuronal homeostasis and energy metabolism highly express GLT-1, GLAST, Kir4.1, GS, LDHA, and connexins 30/43. In contrast, reactive astrocyte subsets exhibit elevated expression of C3, C4b, Serping1, H2-T23, H2-D1, Lcn2, Fkbp5, Srgn, and Amigo2, and can either promote or attenuate neuroinflammation and RGC loss [18]. Notably, astrocyte responses to glaucomatous injury are heterogeneous. Optic nerve head astrocytes show early upregulation of Gulp1 and Lgals3, accompanied by enhanced phagocytic and antioxidative activities that preserve axonal integrity, whereas adjacent astrocytes may remain quiescent or adopt harmful phenotypes [20,21].

This functional diversity extends to metabolic support: astrocytes maintain the bioenergetic microenvironment of RGCs via region-specific expression of metabolic enzymes and transporters, including glutamine synthetase (GS), pyruvate dehydrogenase, GLT-1/GLAST, and monocarboxylate transporters, thus directly regulating neuronal survival under stress [6]. Additionally, astrocyte-mediated inflammatory responses are highly context-dependent, with some subtypes amplifying neuroinflammation and others exerting immunomodulatory or neuroprotective effects [61]. The recognition of this functional heterogeneity is crucial for understanding the complex interplay between astrocyte subpopulations and RGC fate in glaucoma and highlights the potential for targeted therapeutic strategies that modulate specific astrocyte functions to preserve vision [20,61,62].

### 2.3. Special Characteristics of Retinal Astrocytes: Comparison with Other CNS Regions

Retinal astrocytes exhibit distinct anatomical, functional, and molecular profiles relative to other CNS astrocytes. [62]. In terms of morphology, retinal astrocytes are mainly found in the nerve fiber layer, particularly around the optic nerve head and, to a lesser extent, in the peripheral retina, displaying a radial arrangement that is not present in the mid-retina [63]. Furthermore, retinal astrocytes are deeply involved in neurovascular coupling and BRB maintenance, forming intricate interactions with both the vasculature and neuronal elements [63,64]. The expression of key functional proteins such as aquaporin 4 (AQP4) and connexin 43 (Cx43) is regionally regulated in retinal astrocytes, with minimal AQP4 in the lamina cribrosa and dynamic changes in Cx43 expression under stress conditions, reflecting unique regional phenotypes not observed in brain astrocytes [18,65]. Additionally, retinal astrocytes contribute to specialized physiological processes such as energy metabolism, axonal support, and modulation of neuronal excitability, often in concert with Müller glia, and display distinct responses to injury and metabolic stress compared to astrocytes in other CNS regions [6,66].

## 3. Molecular Mechanisms of Retinal Ganglion Cell Damage in Glaucoma

### 3.1. Acute Ocular Hypertension and RGC Apoptosis

Acute elevation of IOP is a key feature of acute glaucoma, leading to a series of harmful events that ultimately result in the apoptosis of RGCs. When IOP rises suddenly, it causes retinal ischemia and hypoxia, which disrupts the metabolic support to RGCs and triggers oxidative stress due to the excessive production of reactive oxygen species (ROS) [19]. This oxidative environment not only inflicts direct damage on cellular components but also activates redox-sensitive signaling pathways, including HIF-1α, NF-κB, and MAPKs, which further intensify inflammation and cell death [67]. Histopathological studies in animal models have consistently shown that acute ocular hypertension leads to significant retinal edema, disorganization of RGC layers, nuclear swelling, and an increase in apoptotic markers, as demonstrated by TUNEL staining and a decrease in RGC survival [68].

In addition, the activation of microglia and neuroinflammation are notable consequences following an acute rise in IOP, with activated microglia releasing pro-inflammatory cytokines and engaging in the phagocytosis of apoptotic RGCs, thereby exacerbating neuronal loss [69,70]. Various pathways of cell death, including apoptosis, necroptosis, pyroptosis, and the newly identified PANoptosis, have been documented, highlighting a complex interaction of regulated cell death mechanisms in response to ischemic and oxidative stress [71,72]. Therapeutic strategies aimed at addressing oxidative stress, inflammation, and specific apoptotic pathways—such as inhibiting NOX4, using neuroprotective agents like carnosic acid, melatonin, and resveratrol, and modulating microglial activation—have demonstrated effectiveness in reducing RGC apoptosis and preserving the structure and function of the retina in experimental models [19,68,73].

### 3.2. Proteomic Insights into Molecular Changes

Proteomic analyses have greatly enhanced our understanding of the molecular changes associated with RGC injury in glaucoma, such as fatty acid-binding protein 7 (FABP7), caveolin-1 (Cav-1), galectin-1 (Gal-1), S100 calcium-binding protein A6 (S100a6), and visinin-like protein-1 (VILIP) [74]. A recent study utilizing mass spectrometry and an acute ocular hypertension model found significant alterations in the levels of FABP7 and Cav-1 following retinal ischemic injury. Furthermore, the study also noted changes in the levels of Gal-1, S100a6, and VILIP, proteins that have been linked to neuronal ischemia and neurodegeneration. The dysregulation of these proteins in the glaucoma model underscores the intricate relationships between metabolic disturbances, neuroinflammation, and calcium homeostasis in RGC injury [74].

### 3.3. The Role of Neuroinflammation and Fatty Acid Metabolism

Neuroinflammation and disturbances in fatty acid metabolism are closely intertwined processes that synergistically contribute to RGC injury in glaucoma. Neuroinflammation, primarily mediated by activated microglia and astrocytes, has emerged as a central mechanism in glaucomatous neurodegeneration [75,76]. Recent studies have demonstrated that elevated IOP and other glaucomatous insults trigger glial activation, leading to the release of pro-inflammatory cytokines, chemokines, and ROS, which exacerbate RGC loss [77,78]. Parallel to these inflammatory changes, metabolic dysregulation—particularly in lipid and fatty acid metabolism—has been observed in the glaucomatous retina. Proteomic analyses in models of acute ocular hypertension, a key feature of glaucoma, reveal significant alterations in proteins involved in fatty acid metabolism, such as FABP7, which is also implicated in ocular inflammatory signaling [74]. Disrupted fatty acid metabolism can directly affect cellular energy homeostasis and membrane integrity, rendering RGCs more susceptible to injury. Moreover, metabolic dysregulation can potentiate neuroinflammatory responses, as aberrant lipid profiles and the accumulation of specific fatty acids can activate glial cells and promote the production of inflammatory mediators [79]. The crosstalk between neuroinflammation and fatty acid metabolism is further exemplified by findings that glial cells, especially astrocytes, modulate both inflammatory and metabolic pathways in response to glaucomatous stress, influencing RGC survival [80]. Notably, interventions targeting either neuroinflammation or metabolic dysfunction—such as the use of anti-inflammatory agents, antioxidants, or metabolic modulators—have shown promise in attenuating RGC loss, underscoring the therapeutic potential of disrupting this pathological synergy [78,79].

## 4. Metabolic Reprogramming of Astrocytes

### 4.1. Remodeling of Energy Metabolic Pathways

Astrocyte energy metabolism reprogramming—integrating glycolysis, oxidative phosphorylation (OXPHOS), and fatty acid oxidation (FAO)—is a key mediator of RGC survival in glaucoma [81]. Glaucomatous stress triggers adaptive shifts: elevated IOP upregulates astrocyte OXPHOS/mitochondrial translation [21], while enhanced glycolysis and lactate release support neuronal energy supply under neuroinflammatory/hypoxic conditions [82,83,84]. However, pathological alterations—mitochondrial dysfunction (impaired OXPHOS, disrupted dynamics) [85] and defective FAO (e.g., reduced long-chain acylcarnitines, downregulated CPT1A) [86]—drive RGC vulnerability. This metabolic crosstalk underscores astrocyte metabolism as a potential therapeutic target to preserve RGC function in glaucoma, linking metabolic pathway dysregulation to disease progression.

### 4.2. Fatty Acid Metabolism and the Regulatory Role of FABP7

As a specialized lipid chaperone, FABP7 mediates the translocation of long-chain fatty acids (e.g., oleic acid, docosahexaenoic acid, palmitic acid) across subcellular compartments. It binds diverse fatty acid species via distinct conformations to enable targeted delivery, thereby modulating transcriptional programs associated with lipid metabolism, energy homeostasis, and inflammation [87,88,89]. Highly enriched in astrocytes and neural progenitor cells, FABP7 regulates metabolic cascades via interactions with key enzymes (e.g., ATP-citrate lyase) to modulate histone acetylation and gene expression [90]; its upregulation further promotes lipid droplet biogenesis and transcription of cell growth/immune signaling genes [91,92]. Dysregulated FABP7 expression in acute ocular hypertension and neurodegenerative disorders correlates with fatty acid metabolic perturbations and exacerbated inflammation, highlighting its central regulatory role in disease progression [74,93].

### 4.3. The Impact of Metabolic Reprogramming on RGC Survival

RGC survival in glaucoma depends on balancing energy supply and demand, with mitochondria as key metabolic hubs. Stress-induced (hypoxia, elevated IOP) metabolic shifts from OXPHOS to glycolysis—regulated by hypoxia-inducible factors (HIFs) and mitophagy [94,95]—initially sustain RGCs but drive apoptosis when chronic [96], worsened by disrupted nuclear-mitochondrial crosstalk and energy insufficiency [97]. Importantly, metabolic-targeting agents (e.g., WAY-100635) preserve RGC viability, axon regeneration, and visual function in experimental glaucoma [98]. This links RGC metabolic dysregulation to disease progression and positions targeted metabolic reprogramming as a promising translational strategy for neuroprotection in glaucoma and other optic neuropathies [95,98].

## 5. Astrocyte-Mediated Neuroinflammatory Response

### 5.1. Secretion of Inflammatory Cytokines and Signal Transduction

Astrocytes are key regulators of CNS neuroinflammation, and their role in glaucomatous RGC injury has gained increasing recognition—indeed, abrogating the formation of neuroinflammatory reactive astrocytes confers RGC protection in a murine glaucoma model [22]. Pathological stimuli trigger astrocyte reactivity and the secretion of pro-inflammatory cytokines (e.g., IL-1β, TNF-α) [99,100], which in turn activate the NF-κB, PI3K/Akt, MAPK, JAK/STAT3, and Notch-PI3K-Akt signaling pathways to amplify neuroinflammation and neurotoxicity [99,101,102,103]. Astrocyte heterogeneity underpins context-dependent pathway activation [99,104]; meanwhile, elevated TNF-α (which disrupts RGC calcium homeostasis) and IL-1β (which sustains chronic neuroinflammation) exacerbate RGC degeneration in glaucoma [105,106]. Targeting these pro-inflammatory pathways (e.g., via NF-κB or PI3K/Akt inhibition) alleviates astrocyte-mediated neurotoxicity and affords RGC protection [107,108].

### 5.2. The Link Between Neuroinflammation and RGC Damage

Neuroinflammation is a central driver linking retinal injury to progressive RGC degeneration in glaucoma and related optic neuropathies. Pathological insults (elevated IOP, ischemia-reperfusion, traumatic optic nerve damage) consistently activate resident microglia and recruit peripheral immune cells to the retina, which secrete pro-inflammatory cytokines (TNF-α, IL-1β, IL-6) that directly induce RGC apoptosis and impair neuronal function [76,109,110]. The NLRP3 inflammasome, a key inflammatory regulator, is significantly upregulated in glaucoma, mediating IL-1β maturation to amplify the neurotoxic microenvironment [111,112]. Inflammatory cascades further promote oxidative stress and mitochondrial dysfunction [113,114], disrupt the BRB, and perpetuate chronic immune cell infiltration, collectively accelerating RGC loss [115,116]. Mechanistically, targeting neuroinflammatory pathways (e.g., HMGB1, NLRP3, TLR4 inhibition) or administering anti-inflammatory agents (natural products, small-molecule inhibitors, cytokine blockers) reduces microglial activation, lowers cytokine levels, and enhances RGC survival in preclinical models [78,112,117,118,119].

## 6. Astrocytes and Retinal Microenvironment Homeostasis

### 6.1. Maintenance of the Blood-Retinal Barrier and Extracellular Matrix

Astrocytes play a critical role in sustaining BRB function—essential for retinal homeostasis and RGC survival—via end-feet interactions with retinal blood vessels and secretion of factors such as glial cell line-derived neurotrophic factor (GDNF), transforming growth factor-β (TGF-β), basic fibroblast growth factor (bFGF) and angiopoietin 1 (ANG1) regulating endothelial tight junctions [120]. Under oxidative stress or neuroinflammation, astrocyte activation exerts dual effects: upregulating ZO-1, occluding, claudin-5proteins to support the BRB or secreting pro-inflammatory mediators that promote endothelial dysfunction and barrier breakdown [121]. Interventions with the nonsteroidal anti-inflammatory drug bromfenac—which modulate astrocyte reactivity or astrocyte-derived pathways—have shown efficacy in preserving BRB integrity and reducing RGC loss in experimental glaucoma and retinopathies [119]. Extracellular matrix (ECM) remodeling alters the biomechanical properties including strength, stiffness, structural rigidity, compliance, and nutrient diffusion of the optic nerve head and peripapillary sclera, accompanied by enhanced resident astrocyte reactivity that contributes to optic neuropathy [122]. Targeting astrocyte reactivity or related pathways (pAKT/AKT, TGF-β2/CCN2/CTGF, Nrf2-ARE) maintains BRB and ECM integrity, reducing RGC degeneration in glaucoma and retinopathies [15,16,27], underscoring astrocytes as key regulators of retinal vascular and ECM homeostasis in glaucoma. These findings suggest that astrocyte-targeted therapies may offer novel strategies to protect against BRB and ECM disruption and subsequent neurodegeneration in glaucoma.

### 6.2. Ion and Neurotransmitter Homeostasis

Astrocytes orchestrate CNS ion and neurotransmitter homeostasis via specialized channels, transporters, and receptors: inwardly rectifying K+ channels mediate extracellular K+ spatial buffering to prevent neuronal hyperexcitability [123], while excitatory amino acid transporters (EAATs) clear synaptic glutamate (converting it to glutamine for neuronal reuse) to mitigate excitotoxicity [124,125]. They also modulate GABA levels to sustain synaptic stability [126,127], with functional heterogeneity driven by region-specific ion channel/transporter expression [123,128]. In glaucoma and other neurodegenerative conditions, pathological stress impairs astrocytic regulatory capacity [129,130], disrupting K+/glutamate dynamics and exacerbating RGC vulnerability—underscoring the mechanistic link between astrocyte dysfunction and neuronal injury.

## 7. Application of Proteomics in Glaucoma Research

### 7.1. Mass Spectrometry Techniques and Quantitative Proteomics

Mass spectrometry (MS) and quantitative proteomics techniques—including tandem mass tag (TMT) and isobaric tags for relative and absolute quantification (iTRAQ)—enable sensitive, reproducible multiplexed protein quantification (thousands per MS run), transforming retinal research in RGC injury and glaucoma [28,34,131]. Mechanistically, TMT-based quantitative proteomics in normal tension glaucoma (NTG) retinal samples (over 6000 proteins identified) uncovered dysregulation in protein synthesis, energy metabolism, and autophagy-lysosome pathways, providing insights into NTG progression [34]. Similarly, iTRAQ-MS analysis of astrocytes under mechanical stretch (mimicking glaucomatously elevated IOP) revealed activation of Wnt/β-catenin, NF-κB, and PI3K-Akt pathways—key mediators of astrocyte reactivity and subsequent RGC injury [35]. With advances in sample preparation, data acquisition, and computational analysis, MS-based proteomics increasingly facilitates exploration of astrocyte diversity and metabolic changes, which are pivotal for elucidating glaucoma’s underlying mechanisms [132,133].

### 7.2. Functional Analysis of Key Proteins

Gene Ontology (GO) and Kyoto Encyclopedia of Genes and Genomes (KEGG) analyses underpin the mechanistic understanding of protein networks in glaucomatous RGC injury, particularly regarding astrocyte heterogeneity and metabolic dysregulation. By integrating transcriptomic and proteomic profiling (multi-omics), researchers generate large datasets to functionally annotate and map pathways of differentially expressed proteins (DEPs) in glaucomatous retinas [29,36]. Mechanistically, comparative proteomics of RGCs under stress (glutamate excitotoxicity, optic nerve crush) uncovers DEPs—some overlapping, some distinct—that converge on immune system activation and metabolic disturbances, key drivers of retinal injury [36]. Pathway enrichment via GO/KEGG highlights NF-κB and Hippo pathways as critical in primary open-angle glaucoma, with proteins Bcl3 and Edn2 identified as central mediators; integrated single-cell RNA sequencing, ATAC sequencing, and functional enrichment further validate that Bcl3/Edn2 upregulation correlates with immune cell infiltration and neuroinflammatory responses [29]. In acute retinal injury models, GO/KEGG analyses reveal transcriptional patterns: upregulated genes cluster in immune-related pathways, while downregulated genes associate with synaptic maintenance and neurogenesis—reflecting the balance between immune regulation and neuronal health. Additionally, protein-protein interaction (PPI) network analysis, paired with GO/KEGG, identifies hub genes like Ccl5, which act as central nodes in functional networks and underscore the utility of these tools for dissecting glaucomatous injury mechanisms [134] (Table 2).

## 8. Astrocyte–Retinal Ganglion Cell Interactions

### 8.1. Metabolic Support and Neurotrophic Factors

Astrocytes are indispensable for RGC survival and function, especially under glaucomatous stress. They supply metabolic support—most notably lactate, a critical neuronal fuel when glucose metabolism is impaired—while metabolic dysregulation in the glaucomatous retina is a key driver of RGC degeneration [79]. Astrocytes also secrete neurotrophic factors, including brain-derived neurotrophic factor (BDNF), nerve growth factor (NGF), glial cell line-derived neurotrophic factor (GDNF), and mesencephalic astrocyte-derived neurotrophic factor (MANF), which directly promote neuronal survival and regeneration [135,136]. Studies confirm that astrocytic dysfunction or depletion of these factors increases RGC vulnerability to glaucomatous injury, whereas their overexpression or supplementation confers robust neuroprotection [23,137,138]. For example, astrocytic BDNF and NGF are upregulated via the NF-κB pathway in response to injury or inflammatory signals such as TNF-α, reflecting a dynamic injury-responsive neuronal support mechanism [139]. Astrocyte-derived secreted phosphoprotein 1 (SPP1, also known as osteopontin) enhances RGC survival, mitochondrial function, and energy metabolism, reinforcing metabolic support [34]. Collectively, these findings highlight astrocytes’ critical role in providing metabolic substrates and neurotrophic factors—essential for RGC resilience—suggesting targeting these mechanisms as a promising glaucoma therapeutic strategy.

### 8.2. Mechanisms of Intercellular Signaling

Astrocyte-retinal cell intercellular signaling—mediated by gap junctions and extracellular vesicles (e.g., exosomes)—is fundamental to glaucomatous RGC injury pathophysiology [18]. Gap junctions, primarily composed of connexins such as Connexin43 (Cx43), enable cytoplasmic exchange of ions and small molecules to coordinate glial responses and neuronal metabolic support. In experimental glaucoma, elevated IOP downregulates astrocytic Cx43, correlating with astrocyte activation and optic nerve head plasticity alterations. This Cx43 reduction is regulated by the Rac1/PAK1 pathway; its pharmacological or genetic modulation impacts astrocytic ATP release and RGC survival. Specifically, Rac1 inhibition enhances Cx43-mediated ATP release, promoting RGC survival via adenosine A3 receptor upregulation—highlighting neuroprotective astrocyte-neuron crosstalk [24].

Exosomes and other extracellular vesicles also mediate critical intercellular signals in glaucomatous degeneration [140]. Astrocyte-derived exosomes increase primary neurite length in vitro, while exosomes from Loxl1-deficient optic nerve head astrocytes lack this trophic effect. This suggests Loxl1 dysfunction and impaired elastin synthesis during reactive astrocytosis may disrupt neuron-glia signaling in glaucoma [30]. Microglia-derived exosomes, particularly under hydrostatic pressure elevation mimicking glaucoma, propagate pro-inflammatory signals, enhance microglial activation, and induce RGC death and oxidative stress in vitro and in vivo. Acting autocrinally and paracrinally, these exosomes sustain and amplify neuroinflammation, exacerbating retinal degeneration [141]. Astrocytes also engage in complex crosstalk with neurons and other glial cells via multiple signaling modalities, including chemical messengers (glutamate, ATP, and D-serine), calcium waves, and lipid mediators (prostaglandins, sphingosine 1-phosphate, endocannabinoids, and lysophosphatidic acid); gap junctions propagate intercellular calcium waves to modulate synaptic activity and neurotransmitter homeostasis [142,143]. These findings underscore the multifaceted mechanisms of astrocyte-mediated intercellular signaling—through both direct cell-cell contacts and extracellular vesicle-mediated pathways—in the regulation of RGC health and the progression of glaucomatous injury.

## 9. Astrocyte-Targeted Strategies in Glaucoma Treatment

### 9.1. Prospects for the Application of Metabolic Modulators

Metabolic pathway targeting—encompassing fatty acid metabolism, glycolysis, nicotinamide adenine dinucleotide (NAD^+^) homeostasis, and iron metabolism—emerges as a promising glaucoma treatment, centered on RGC/astrocyte metabolic dysfunction and RGC injury in neurodegeneration. Mechanistically, mitochondrial dysfunction and altered lipid metabolism are integral to glaucoma pathogenesis, with genetic/proteomic studies linking mitochondrial lipid metabolism genes to primary open-angle/angle-closure glaucoma susceptibility [38]. NAD^+^ metabolism, governed by CD38, regulates energy homeostasis and RGC survival; CD38 inhibition or nicotinamide supplementation restores NAD^+^-dependent neuroprotective signaling [144]. Iron chelators (e.g., Deferiprone) inhibit ferroptosis (iron-dependent, lipid peroxidation-driven cell death) to protect RGCs [37], while HIFs -mediated glucose transporter regulation under ocular hypertension-induced hypoxia supports glycolytic targeting for meeting RGC metabolic demands [41,42]. Nicotinamide further enhances trabecular meshwork mitochondrial function to reduce IOP [39,40]. These converging pathways highlight metabolic modulators as targeted glaucoma therapeutics.

### 9.2. Anti-Inflammatory Therapy and Neuroprotection: Strategies to Inhibit Inflammatory Responses and Enhance Astrocytic Neuroprotective Functions

Inflammation contributes to RGC degeneration in glaucoma, with astrocytes acting as central regulators of neuroinflammatory and neuroprotective responses—their reactivity is a critical determinant of RGC survival. Mechanistically, anti-GFAP monoclonal antibodies inhibit astrogliosis, suppress neuroinflammation, and protect RGCs by downregulating p38 MAPK, NF-κB, and NLRP3 inflammasome pathways, and reducing pyroptosis [43]. Astrocyte-derived lipoxin B4 (LXB4) exerts direct neuroprotection on RGCs and modulates astrocyte activity under inflammatory or ocular hypertensive conditions [31]. The IL-33/ST2 axis shifts astrocyte phenotypes toward neuroprotection, while astrocytic SPP1 deletion increases RGC vulnerability—vitamin C upregulates SPP1 to enhance RGC survival [44,45]. cFLIP (FLICE-like Inhibitory Protein, Caspase-8 FADD-like Apoptosis Regulator) limits glia-driven neuroinflammation in ocular hypertension [25], and P2X7R stimulation is necessary and sufficient for IOP-induced pan-, A1-, and A2-type astrocyte activation [33].Additionally, Glucagon-Like Peptide-1 receptor agonists and natural compounds (naringenin, crocetin, baicalein) mitigate inflammation/oxidative stress by regulating astrocyte/microglial activation [26,32,107,108], with astrocyte polarization (A1→A2 shift) emerging as a key therapeutic target [26,145] (Table 3).

## 10. Emerging Theories and Research Progress

### 10.1. Astrocyte Subtypes Revealed by Single-Cell Sequencing

The rapid advancement of single-cell omics—particularly single-cell RNA sequencing and single-nucleus RNA sequencing (snRNA-seq)—has deepened our understanding of astrocyte diversity in the CNS, with critical insights into glaucoma-relevant regions (retina, ONH). These technologies reveal distinct astrocyte subtypes that are regionally and functionally heterogeneous in both healthy and diseased CNS tissues (including retina/ONH, primary sites of RGC injury in glaucoma) [146]. Beyond the well-characterized A1/A2 reactive astrocyte states, single-cell analyses uncover additional subdivisions defined by gene expression patterns, metabolic activity, and proliferation—especially under neurodegenerative stress [147]. For instance, single-cell RNA sequencing studies show that Abca1 deficiency in aged Glia-KO mice induces astrocyte-driven inflammation and increases the susceptibility of specific RGC clusters to excitotoxicity [17]. In glaucoma, single-cell omics further demonstrate that ONH and retinal astrocyte populations are not uniform; instead, they exhibit region-specific and time-dependent transcriptional changes in response to elevated IOP and RGC stress-alterations with profound implications for neuroprotection and neurodegeneration [148].

### 10.2. New Mechanisms of Metabolism-Inflammation Crosstalk: Molecular Mechanisms of Metabolite Regulation of Inflammatory Responses

Astrocyte metabolic reprogramming and inflammation are central to understanding RGC injury in glaucoma and other neurodegenerative diseases. Mechanistically, pyruvate kinase M2 (PKM2) regulates astrocyte inflammatory responses by translocating to the nucleus to promote cytokine production—this process is tightly modulated by tripartite motif-containing 21 (TRIM21). Inhibition of PKM2 nuclear translocation or TRIM21 knockdown significantly reduces astrocyte proliferation and neuroinflammation [149]. Nicotinamide phosphoribosyltransferase (NAMPT) sustains astrocyte NAD^+^ metabolism, a key determinant of energy homeostasis; however, neuroinflammation upregulates CD38, triggering NAD^+^ depletion and pro-inflammatory gene induction. CD38 inhibition reverses NAD^+^ loss, suppresses inflammatory responses, and impedes monocyte recruitment, thereby alleviating CNS inflammation [150]. Beyond this, glycolytic byproducts (e.g., lactate) modulate immune cell function and local tissue pH, indirectly influencing inflammatory processes and neuronal damage [151], while astrocytic lipid droplets and β-oxidation act as critical links between metabolic stress, oxidative stress, and inflammation [152]. Collectively, these findings reveal the intricate crosstalk between metabolic enzymes and metabolites in regulating astrocyte inflammation, underscoring novel mechanistic targets for glaucomatous neurodegeneration.

## 11. Future Research Directions and Challenges

### 11.1. Multi-Omics Integrated Analysis

High-throughput approaches (single-cell RNA-seq, bulk RNA-seq, ATAC-seq) have uncovered dysregulated genes, chromatin accessibility, and signaling pathways in glaucoma. Furthermore, multi-omics strategies further define proteomic and metabolomic alterations in ocular fluids. NanoDSF-based tear proteomics revealed primary open-angle glaucoma-specific denaturation signatures linked to proteomic, lipidomic, and metallomic regulation [153]. Single-cell transcriptomics delineated RGC subtype heterogeneity and injury-responsive transcriptomic signatures.

Collectively, multi-omics integration enables systematic dissection of molecular net- works underlying glaucomatous neurodegeneration, supporting the identification of context-specific therapeutic targets and the development of mechanism-driven precision medicine [154].

### 11.2. Clinical Translation and Personalized Therapy

The translation of glaucoma research into clinical practice is hindered by multiple challenges, particularly astrocyte heterogeneity and RGC injury. Notably, human in vitro models (e.g., organoids, assembloids) provide physiologically relevant platforms to dissect the cellular mechanisms underlying RGC degeneration, which are difficult to recapitulate in conventional cell cultures or animal models. While promising approaches such as nanotechnology, gene therapy, and focused ultrasound enhance drug penetration across the BRB), issues including off-target effects and systemic toxicity impede their clinical translation [155]. However, translation of animal findings to humans remains challenging due to inherent species differences in retinal physiology, disease progression, and therapeutic response, which frequently impede direct clinical translation of preclinical results. Furthermore, the heterogeneity of disease etiologies, patient genetic backgrounds, and environmental exposures necessitates the development of robust biomarkers for patient stratification and disease monitoring—an essential prerequisite for the realization of personalized medicine [156].

## 12. Conclusions

Astrocytes are now recognized as pivotal regulators of RGC injury in glaucoma, shaping RGC survival via diverse, context-dependent responses—their dual role in exacerbating or alleviating neural damage is dictated by the cellular microenvironment and signaling cues. Underlying mechanisms center on fatty acid metabolism and neuroinflammatory pathways, which engage in intricate crosstalk with other retinal cell populations. High-throughput proteomics has provided advanced insights into the functional alterations of astrocytes associated with glaucoma, uncovering novel regulatory pathways and potential biomarkers. Resolving conflicting findings requires critical analysis, as experimental model variability and inherent astrocyte heterogeneity drive divergent outcomes—underscoring the need for standardized models and single-cell resolution techniques. Targeting astrocyte metabolism (e.g., fatty acid oxidation) and neuroinflammatory pathways represents a promising glaucoma therapeutic strategy, with fatty acid oxidation modulation showing considerable preclinical potential.

## Figures and Tables

**Table 1:** Overview of key findings for the astrocyte role in various glaucoma models.

Experimental Model Type	Species, Strain & Age	Astrocyte Observation Location	Key Points	References
Inherited Glaucoma Model	Mice, DBA/2J, age not specified	Retina, optic nerve head	Astrocyte activation/gliosis upregulates MMP9, causing axonal injury, ECM remodeling and ONH cupping.	[1]
	Mice, 129/Sv--C57BL/6--CD1 mixed background, 12 weeks	Optic nerve, Optic nerve head	DCN antagonizes TGF-β/CTGF through pAKT/AKT; DCN-KO mice exhibit elevated TGF-β1/β2, CTGF in optic nerve astrocytes.	[15]
	Mice, C57BL/6J (βB1-CTGF1 transgenic), 1 and 2 months	Optic nerve head, specifically the glial lamina	Increased CCN2/CTGF in ONH astrocytes exacerbates ONH injury via ECM and cytoskeletal changes.	[16]
	Mice, C57BL/6J (Glia-KO), DBA/1J (conventional KO), 12–18 months	Retina, optic nerve head, optic nerve	ABCA1 deficiency induces astrocyte dysfunction (hypertrophy → GFAP reduction), neuroinflammation, abnormal cholesterol metabolism, and RGC non-cell-autonomous damage.	[17]
Laser-induced ocular hypertension Model	Rats (strain not specified), adult	Retinal Nerve Fiber Layer, Optic nerve head (particularly the Lamina Cribrosa region)	GFAP upregulation and astrocyte activation promote pro-inflammatory cytokine release, exacerbating RGC damage.	[13]
Acute Optic Nerve Injury Model	Mice, C57BL/6J, age not specified	Retina	Injury-induced Cx43 upregulation in reactive astrocytes exerts harmful effects via hemichannel activity in disease pathogenesis.	[18]
	Mice, C57BL/6J, male, 6–8 weeks	Whole retinal mounts and the mid-peripheral regions of retinal longitudinal sections.	GLX351322 (NOX4 inhibitor) improves retinal function by inhibiting oxidative stress, inflammation, glial activation, and retinal cell damage.	[19]
Microbead Occlusion Glaucoma Model	Mice, C57BL/6, B6.hGFAPpr::EGFP, 13 weeks (young adult), 40 weeks (middle-aged)	Optic nerve head (specifically, the unmyelinated glial lamina region)	IOP elevation induces astrocyte reactive heterogeneity (phagocytosis ↑, hyperplasia); glaucomatous/aged ON show matching astrocyte gene/morphology traits.	[20]
	Mice, C57Bl/6 mice, and GFAP-Cre mice, 3–4 months	Optic nerve head, optic nerve	Ocular hypertension-induced early astrocyte response: spatiotemporal heterogeneity, adaptive metabolic shift (no neuroinflammation) for homeostasis.	[21]
	Mice, C57Bl/6J and IL-1α/TNF/C1q triple-knockout mice; adult, female,	Retina, optic nerve head	IL-1α/TNFα/C1q induce neurotoxic reactive astrocytes, driving RGC loss; pathway ablation in cKO mice suppresses astrocyte toxicity and promotes RGC survival.	[22]
	C57BL/6, SPP1Cko, 3–5 months and 16 months	Optic nerve head, Proximal optic nerve, Retina	High IOP: reactive astrocytes (SPP1 ↑, A1 markers ↑, dysfunction) → accelerated RGC loss.	[23]
	Mice, C57BL/6, Rac1(flox/flox) mice, adult	Optic nerve head, Retinal nerve fiber layer	Elevated IOP activates astrocytes and inhibits Cx43 via Rac1/PAK1 pathway; Rac1 targeting reverses this, enhances Cx43-mediated ATP release and protects RGCs.	[24]
	Mice, C57BL/6J, cFLIP-Cko, adult	Retina, Optic nerve	cFLIP-specific knockout astrocytes show reduced pro-inflammatory and increased anti-inflammatory cytokines, protecting RGCs.	[25]
	Rat, Brown Norway, weight 250–320 g (approximately 8–10 weeks)	Retina (areas distant from retinal blood vessels)	Ocular hypertension increases astrocyte fractal dimension; semaglutide (GLP-1 agonist) reverses this OHT-induced elevation.	[26]
In vitro 3D hydrogel-based compression strain model	Mice, C57BL/6J, 6–8 weeks	In vitro 3D Hydrogel (Core and Periphery regions)	Compressive strain reduces F-actin coverage, upregulates GFAP/HIF-1α, induces hypoxia/oxidative phosphorylation transcriptomic changes, increases fibronectin deposition, and causes astrocyte dysfunction.	[27]
Episcleral-vein-cauterized model (Subconjunctival injection of 5-fluorouracil)	Rat, Sprague Dawley, 7–8 weeks old	Optic nerve head, Lamina cribrosa	Early ONH astrocytes: neuroprotective A2 phenotype; prolonged high IOP shifts to neurotoxic A1 phenotype.	[28]
Excitotoxicity Models	Rat, Sprague Dawley, 6–8 weeks	Retina	This model upregulates astrocyte Edn2; astrocyte activation links to NF-κB/Hippo pathways, promoting RGC death.	[29]
Anterior chamber perfusion induced high intraocular pressure model	Rat, Sprague Dawley, 6–8 weeks	Retina, Optic nerve head	This model induces ONH neuroinflammation; astrocyte-derived Edn2 acts paracrinely to affect vessels, recruit immune cells and exacerbate damage.	[29]
Equibiaxial mechanical strain model	Primary optic nerve head astrocytes from Sprague Dawley rats, Cells at passages 5 to 26	Direct observation	Reactive astrocytes show cytoskeletal remodeling (actin shortening), GFAP/ROS upregulation, LOXL1/elastin downregulation, and exosome neurotrophic function loss.	[30]
Silicone oil-induced chronic ocular hypertension model	Mice, C57BL/6J, 8 weeks	Retina, Optic nerve	Chronic OHT induces astrocyte reactivity and lipoxin pathway dysregulation; LXB_4_ treatment alleviates reactivity and exerts neuroprotection.	[31]
	Mice, C57BL/6J and Alox5^−^/^−^ (5-LOX-KO), 8 weeks	Retina	Ocular hypertension impairs functional integrity of retinal astrocyte-mediated LXB_4_ circuit.	[32]
Anterior chamber cannula-induced transient intraocular pressure elevation model	Mice, C57BL/6J (wild-type and P2X7^−^/^−^ KO), adult	Retina	P2X7 knockout mice show no significant upregulation of astrocyte activation-related genes under elevated IOP	[33]

This table provides an overview of key findings regarding the role of astrocytes in various glaucoma models, including inherited glaucoma, laser-induced ocular hypertension, acute optic nerve injury, microbead occlusion glaucoma, and other in vitro and in vivo models, utilizing experimental subjects such as mice (e.g., DBA/2J, C57BL/6J strains) and rats (e.g., Sprague Dawley, Brown Norway strains) of different ages (ranging from 6 weeks to 18 months or unspecified). Astrocytes were observed in locations including the retina, optic nerve head (particularly the glial lamina and lamina cribrosa), optic nerve, and in vitro 3D hydrogels, with core observations indicating that astrocyte activation, reactive heterogeneity, phenotypic shifts (e.g., from neuroprotective A2 to neurotoxic A1), and dysregulation of molecules (such as MMP9, TGF-β, Cx43, GFAP, SPP1, Edn2) and pathways (e.g., pAKT/AKT, Rac1/PAK1, NF-κB/Hippo, IL-1α/TNFα/C1q) are closely associated with pathological processes like axonal injury, ECM remodeling, neuroinflammation, abnormal metabolism, and RGC damage or loss in glaucoma. Additionally, interventions including specific inhibitors (e.g., GLX351322, semaglutide, LXB_4_), gene knockout (e.g., DCN-KO, P2X7-KO, cFLIP-Cko), and pathway targeting can alleviate astrocyte dysfunction, inhibit harmful activation, and protect RGCs or improve retinal function.

**Table 2:** Key findings of proteomics.

Research Object	Key Technology	Core Pathways/Molecules	Main Findings	References
Normal tension glaucoma retina	TMT-based quantitative proteomics	Protein synthesis, energy metabolism, autophagy-lysosome pathways	Over 6000 proteins identified; the above pathways are dysregulated and involved in NTG progression	[34]
Astrocytes under mechanical stretch (elevated IOP)	iTRAQ-MS	Wnt/β-catenin, NF-κB, and PI3K-Akt pathways	Pathways are activated, mediating astrocyte reactivity and subsequent RGC injury	[35]
RGCs under stress	Comparative proteomics + multi-omics	Immune system and metabolism-related pathways	Differentially expressed proteins converge on immune activation and metabolic disturbances, key drivers of retinal injury.	[36]
Primary open-angle glaucoma	Proteomics + transcriptomics + single-cell RNA sequencing	NF-κB, Hippo pathways; Bcl3, Edn2 proteins	Upregulation of Bcl3/Edn2 correlates with immune cell infiltration and neuroinflammatory responses.	[29]
Acute retinal injury	GO/KEGG pathway enrichment analysis	Immune pathways, synaptic maintenance, neurogenesis	Immune-related genes are upregulated, while genes for synaptic maintenance and neurogenesis are downregulated.	[29]

NTG: normal tension glaucoma; RGC: retinal ganglion cell.

**Table 3:** Astrocyte-Targeted Therapeutic Strategies in Glaucoma Treatment.

Method	Classification	Mechanism	References
Metabolic modulator-targeted therapy	Iron metabolism regulation	Iron chelators (e.g., deferiprone) protect RGCs by inhibiting iron-dependent lipid peroxidation and ferroptosis.	[37]
	Fatty acid metabolism-targeted	Targeting mitochondrial lipid metabolism–related genes improve metabolic disorders and reduces RGC damage in glaucoma.	[38]
	NAD^+^ homeostasis regulation	Inhibiting CD38 or nicotinamide supplementation restores NAD^+^ homeostasis, improves mitochondrial function, promotes RGC survival, and lowers intraocular pressure.	[38,39,40]
	Glycolysis-targeted	HIF-mediated glucose transporter regulation targets glycolysis to sustain metabolic homeostasis and attenuate RGC injury under high IOP-induced hypoxia.	[41,42]
Anti-inflammatory and neuroprotective therapy	Inhibit astrogliosis and neuroinflammation	GFAP monoclonal antibodies protect RGCs by inhibiting p38 MAPK/NF-κB/NLRP3 pathways, reducing pyroptosis and astrogliosis, and alleviating neuroinflammation.	[43]
	Neuroprotection via astrocyte-derived factors	Astrocyte-derived LXB4 protects RGCs and modulates astrocyte activity under inflammation or high intraocular pressure.	[31]
	Regulate astrocyte phenotypic polarization	Regulating IL-33/ST2 axis polarizes astrocytes to neuroprotective phenotype; vitamin C upregulates astrocytic SPP1 and enhances RGC survival.	[44,45]
	Inhibit glia-mediated neuroinflammation	cFLIP inhibits glia-mediated neuroinflammation and mitigates RGC damage under high IOP; P2X7R blockade prevents astrocyte overactivation induced by high IOP.	[25,33]

RGC, retinal ganglion cell; HIFs, hypoxia-inducible factors; GFAP, glial fibrillary acidic protein; MAPK, mitogen-activated protein kinase; NF-κB, nuclear factor kappa B; NLRP3, NOD-like receptor family pyrin domain containing 3; LXB4, lipoxin B4; IL-33, interleukin 33; ST2, suppression of tumorigenicity 2; SPP1, secreted phosphoprotein 1; cFLIP, FLICE-like inhibitory protein; P2X7R, P2X purinoceptor 7.

## Data Availability

No new data were created or analyzed in the study.
